# Research on Dynamic Response under the External Impact of Paper Honeycomb Sandwich Board

**DOI:** 10.3390/ma17081856

**Published:** 2024-04-17

**Authors:** Lehao Lin, Jingjing Hu, Danyang Li, Gaimei Zhang, Hui Liu, Xiaoli Song, Jiandong Lu, Jiazi Shi

**Affiliations:** School of Printing and Packaging Engineering, Beijing Institute of Graphic Communication, Beijing 102600, China; llh1037581120@163.com (L.L.); hj12345256@163.com (J.H.); 13603120159@163.com (D.L.); liuhui1022@126.com (H.L.); songxiao109@163.com (X.S.); lujiandong@bigc.edu.cn (J.L.); shijiazi@bigc.edu.cn (J.S.)

**Keywords:** honeycomb sandwich panels, finite element method, dynamic impact, simulation prediction

## Abstract

The dynamic mechanical behavior and cushioning performance of honeycomb sandwich panels, which are extensively employed in product cushioning packaging due to their exceptional energy absorption capabilities, were examined using a combination of experimental and numerical methods. Several factors, such as maximum acceleration–static stress, cushioning coefficient–static stress, and other curves, were analyzed under various impact conditions. The simulated stress–strain, deformation modes, cushioning coefficients, and other parameters demonstrate consistency with the experimental results. The acceleration, maximum compression, and cushioning coefficient obtained from the experiment and simulation calculation were 30.68 g, 15.44 mm, and 2.65, and 31.96 g, 14.91 mm, and 2.79, respectively. The results indicate that all error values were less than 5%, confirming the precision and reliability of the model. Furthermore, the model was utilized to simulate and predict the cushioning performance of honeycomb sandwich panels with different cell structures and paper thicknesses. These results provide a solid basis for enhancing the design of subsequent honeycomb element structures.

## 1. Introduction

Lightweight honeycomb sandwich structures have been widely applied in various fields, including furniture, automobiles, ships, and aerospace. The outstanding mechanical properties of these structures have attracted considerable research attention from scholars [1,2,3,4,5]. Presently, a primary focus of research on honeycomb structures revolves around their integration with panels to form sandwich structures [6]. A reduction in mass contributes to a greater specific strength and stiffness of the structure [7,8], thereby significantly enhancing the mechanical properties of the material. Research indicates that honeycomb structures are susceptible to impact loads. In the analysis of the mechanical behavior of honeycomb sandwich structures, the performance and structural parameters of the panel and core materials during dynamic processes are highly important. The cushioning performance of honeycomb sandwich structures subjected to impact loads is a major concern, and there are currently many studies and experiments dedicated to this topic globally. These studies cover various aspects, such as the compressive performance under dynamic impact, structural factors, and deformation modes. Some recent research in this area has focused on the cushioning performance of paper honeycomb sandwich panels [9,10,11,12]. Wang et al. [13] used the Cowper Symonds model and segmented function method to create mechanical models for the platform stress and yield stress of paper honeycomb structures under various strain rates. Wang et al. [14] conducted experiments to determine the impact behavior of paper honeycomb structures. The results show that the thickness and length of the honeycomb cell wall are the key factors affecting cushioning performance. The study revealed that increasing the thickness of the core paper significantly weakened the energy absorption effect. Gu et al. [15] investigated the in-plane uniaxial fracture behavior of honeycomb sandwich panels. They identified three deformation modes under a *Y*-axis load: the non-debonding mode, partial debonding mode, and complete debonding mode. Wang et al. [16] proposed a mathematical model to elucidate the correlation between the energy absorption properties of paper honeycomb and environmental humidity, as well as its structural parameters. The authors conducted a comprehensive simulation study to investigate the energy absorption behavior across four distinct deformation stages of the paper honeycomb. Notably, the model’s validity was confirmed by its alignment with the predicted outcomes. Consequently, this research underscores the model’s potential applicability in optimizing design strategies and material selection for paper honeycomb structures.

Given the substantial time and material expenses involved, experimental methods are not always practical for thoroughly analyzing the mechanical properties of honeycomb structures. Consequently, finite element simulation emerged as a reliable and efficient alternative for conducting detailed analyses in this field [17]. Finite element simulations have been widely adopted in numerous academic studies to explore and analyze the cushioning performance exhibited by honeycomb sandwich panels. Xie et al. [18] studied the dynamic mechanical behavior and properties of Nomex honeycomb sandwich panels under impact loads. Their research investigated the effects of honeycomb core density, punch diameter, and so on. The results highlighted that the thickness of the panels played an important role in increasing the impact strength of the Nomex honeycomb panels, and the density of the honeycomb cores played a key role in determining the stiffness of the structure. Ma et al. [19] introduced the construction of an origami honeycomb and built an analytical model with the help of FEM. It was concluded that the origami honeycomb is superior to conventional honeycomb structures in terms of in-plane compression strength and energy absorption. Sun et al. [20] conducted a study on the effect of adhesive geometry on the impact damage of bonded aluminum sandwich panels utilizing both experiments and finite element simulations. The results revealed a linear relationship between the depth of honeycomb core damage and the height of the adhesive filet, emphasizing the critical role of adhesive geometry in impact damage.

Due to the diverse components and intricate internal structure inherent in honeycomb sandwich panels, ensuring accurate model predictions necessitates extensive calculations. This poses a challenge to the performance and memory requirements of the computers, which, in turn, affects computing efficiency [21,22]. Therefore, further experiments and further studies are needed to obtain an accurate model for the damping properties of all kinds of honeycomb sandwich panels. In this paper, the dynamic mechanical performance of sandwich panels made of paper honeycomb was investigated. In this work, we built an impact model of honeycomb sandwich boards with different drop heights and hammer weights, subsequently validating the accuracy and reliability of these models through dynamic experiments and numerical simulations. Furthermore, few simulations have been used to forecast models in order to gain a larger variety of reference data. Most previous research has employed a mix of experiments and simulations to check the correctness and dependability of model data. This research employed a simulation to predict the buffering performance under different honeycomb cell lengths, and the impact of the change in the wall thickness of the surface layer and the core layer on the cushioning performance was observed. These findings establish a robust foundation for the subsequent optimization design of cellular structures.

## 2. Experimental Methodology

### 2.1. Specimen Description

**Figure 1:**
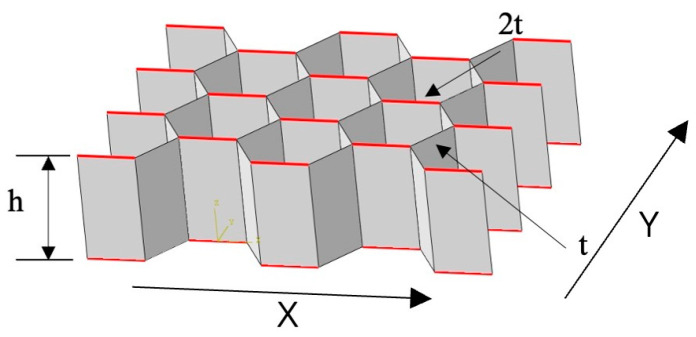
Honeycomb sandwich panel sample.

The sample for testing was a honeycomb sandwich panel, which consisted of two identical surface papers that were bonded to the upper and lower sides of a hexagonal honeycomb core. The height of the sample was determined by adding the height of the honeycomb core to the thickness of the surface papers. The honeycomb core had a fixed height of 30 mm. The thickness of the honeycomb sandwich panel was adjusted by varying the thickness of the honeycomb paper core. Table 1 provides details on the quantification and thickness of the face paper and core paper. Figure 1 shows the individual honeycomb cell of the honeycomb core as a regular hexagon with a side length of 8 mm. A single honeycomb core, neglecting the influence of adhesive, was composed of two honeycomb walls with a thickness of 2 t and four honeycomb walls with a thickness of t. The hexagonal honeycomb exhibited strong anisotropy, with an angle of 120° between the honeycomb walls.

### 2.2. Test Method

**Figure 2:**
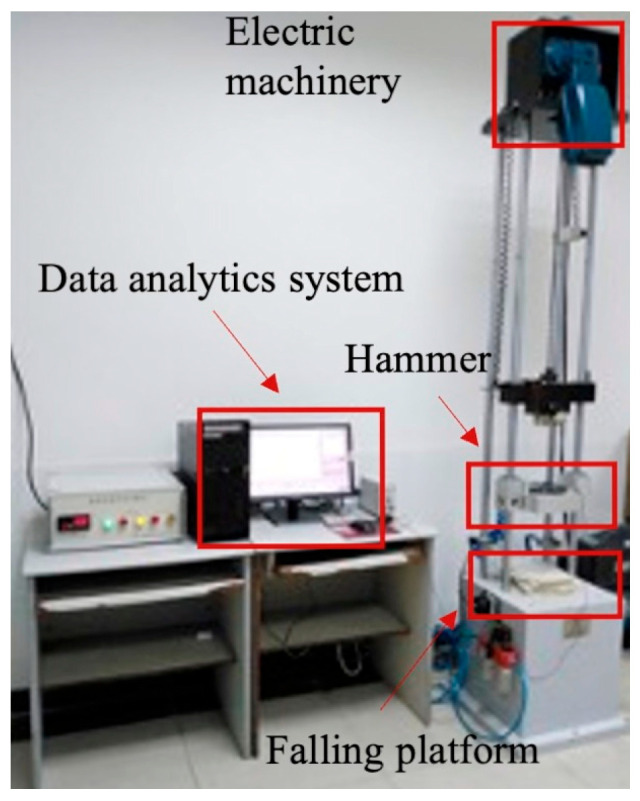
Hammer impact experimental model.

Specimens with a size of 100 mm × 100 mm were fabricated using honeycomb sandwich panels following ASTM-D7766 standards [23]. Before conducting dynamic impact tests, the specimens were conditioned to meet the requirements of standard PN-EN20187:2000 for a duration exceeding 48 h [24]. The impact experiments were carried out using Y52-2/77 (Xian Guangbo Testing Equipment Co., Ltd., Xi’an, China) equipment for testing cushioning materials, as depicted in Figure 2. The composition of the buffer testing apparatus primarily includes a falling platform, an electric machine, a hammer, and data analytics system. Additionally, the experimental device is supplied with pneumatic control attachments to prevent the secondary impact of the punch on the specimen. During the initial impact, test data were generated to ensure the accuracy and reliability of the data. Each experiment was replicated five times under identical conditions. The mass of the impact hammer was adjusted to 7 kg, 8 kg, and 9 kg. The initial energy was autonomously adjusted by altering the drop height by 50 mm. Following each impact, the pulse data acquisition software automatically recorded and analyzed the necessary impact reaction parametric quantities, for instance, the impact acceleration time curve.

### 2.3. Numerical Model

During the preparation of the specimen, the raw paper is treated as a material with directional dependence due to variations in fiber orientation during the production process. In the property module of the Abaqus 2022 software, engineering constants are typically employed to define the material characteristics of the unprocessed paper of honeycomb sandwich panels during the elastic phase. The constitutive relationship of the material in the elastic stage can be represented by the stress–strain curve, as depicted in Formula (1):

(1) $$\left[\begin{array}{c} \varepsilon_{11}\\ \varepsilon_{22}\\ \varepsilon_{33}\\ \gamma_{12}\\ \gamma_{13}\\ \gamma_{23} \end{array}\right]=\left[\begin{array}{cccccc} 1/E_{1} & -\nu_{21}/E_{2} & -\nu_{31}/E_{3} & 0 & 0 & 0\\ -\nu_{12}/E_{1} & 1/E_{2} & -\nu_{32}/E_{3} & 0 & 0 & 0\\ -\nu_{13}/E_{1} & -\nu_{23}/E_{2} & 1/E_{3} & 0 & 0 & 0\\ 0 & 0 & 0 & 1/G_{12} & 0 & 0\\ 0 & 0 & 0 & 0 & 1/G_{13} & 0\\ 0 & 0 & 0 & 0 & 0 & 1/G_{23} \end{array}\right]\left[\begin{array}{c} \sigma_{11}\\ \sigma_{22}\\ \sigma_{33}\\ \sigma_{12}\\ \sigma_{13}\\ \sigma_{23} \end{array}\right]$$

The model uses notations 1, 2, and 3 to represent the *X*, *Y*, and *Z* coordinate axes, respectively. In the formula, *σ_ij_* (Pa) represents the stress component, *ε_ij_*, and *γ_ij_* represent the strain component; *E_i_* (Pa) represents the elastic modulus in the *i* direction, and *G_ij_* (Pa) represents the shear modulus that occurs in the *j* direction of the *i* normal plane deformation. *ν_ij_* represents the Poisson’s ratio of the material and *ν_ij_*/*E_i_* = *ν_ji_*/*E_j_*. The study conducted by Huang et al. [25] on raw paper for honeycomb sandwich panels provides empirical formulas for nine elastic constants, namely:

(2) $$E_{3}=\frac{E_{1}}{200}$$

(3) $$G_{12}=\frac{1}{\frac{4}{E_{45}}-\frac{1}{E_{1}}+2\cdot \frac{v_{12}}{E_{1}}-\frac{1}{E_{2}}}$$

(4) $$G_{13}=\frac{E_{1}}{55}$$

(5) $$G_{23}=\frac{E_{2}}{35}$$

(6) $$\nu_{12}=0.293\sqrt{E_{2}/E_{1}}$$

**Figure 3:**
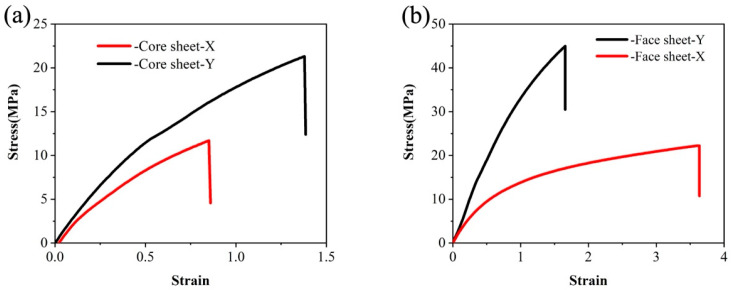
Stress–strain curve of the tensile test: (**a**) core sheet; (**b**) face sheet.

The values of *ν*_13_ and *ν*_23_ are often set to 0.01 [25]. Based on the elastic modulus obtained from the tensile test, the stress–strain curve is shown in Figure 3, and the values of these nine elastic constants can be obtained. The material characteristics of the face paper and core paper are shown in Table 2.

In addition, when defining plastic materials in Abaqus, the actual stress and plastic strain need to be considered. Suppose the nominal stress and strain are obtained in the experiment. In that case, they need to be converted using the following formula, where *σ_n_* (Pa) and *ε_n_* represent the nominal stress and nominal strain, respectively; *σ* (Pa) and *ε* represent the actual stress and true strain, respectively; *ε^pl^* and *ε^el^* represent the plastic strain and elastic strain, respectively; and *E* (Pa) represents the elastic modulus.

(7) $$\sigma =\sigma_{n}\left(1+\varepsilon_{n}\right)$$

(8) $$\varepsilon =ln\left(1+\varepsilon_{n}\right)$$

(9) $$\varepsilon^{pl}=\varepsilon -\varepsilon^{el}=\varepsilon -\frac{\sigma }{E}$$

### 2.4. Finite Element Modeling

Levent Aktay [26] and M Giglio et al. [27] underscored that FEMs for honeycomb sandwich panels can be generally classified into two distinct groups: equivalent homogeneous models and micromechanical models. The former employs the direct application of homogeneous solid units to construct a model, simulating it by assigning parameters with overall mechanical properties. While this method exhibits efficient computational performance, it concurrently neglects the impact of local kernel failure mechanisms on the model. The latter strives to establish a model consistent with the honeycomb panel structure, aiming for a more realistic simulation of alterations in the structural behavior of the honeycomb core material.

**Figure 4:**
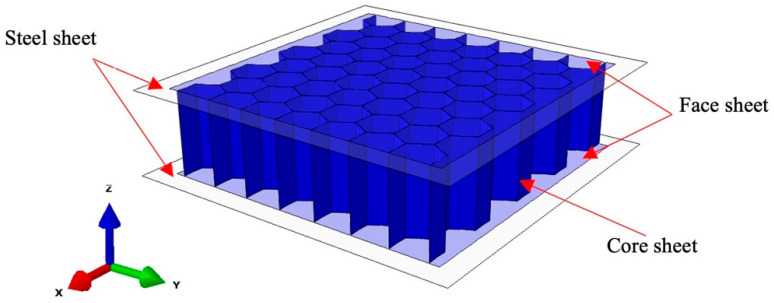
Impact simulation model structure.

As depicted in Figure 4, this article utilized the ABAQUS 2022 finite element software to create a dynamic impact model of honeycomb sandwich panels comprising two main components: a rigid panel and a honeycomb sandwich panel structure. The rigid panel utilizes shell elements with thicknesses of 1 mm and 120 mm in length and width, respectively, using an explicit dynamic solver. To streamline the computational complexity, the model was simplified by excluding the addition of a hammer component. Instead, the velocity of the free-falling body in contact with the honeycomb sandwich panel, just before the application of its drop height, was assigned to the reference point of the upper steel plate. The hammer’s mass was then applied to expedite the model and reduce the calculation time for dynamic display. The reference point on the upper steel plate underwent rotation/displacement constraints, allowing movement solely in the Z direction while being fixed in other directions. The lower steel plate was subjected to completely fixed boundary condition constraints. To replicate the real adhesive layer, binding limitations were applied between the face and core papers, and universal contact was established between the face paper and the steel plate. The contact attribute was configured as hard contact, with a friction coefficient of 0.1, using reasonable grid partitioning to achieve computational convergence. The R3D4 linear quadrilateral element type was used for the rigid plates placed at the top and bottom of the structure. The core layer adopts S4R elements, which can be used for modeling thin or thick shell structures, using the reduced integration method, including hourglass mode control.

## 3. Results and Discussion

### 3.1. Experimental and Simulated Deformation Process

**Figure 5:**
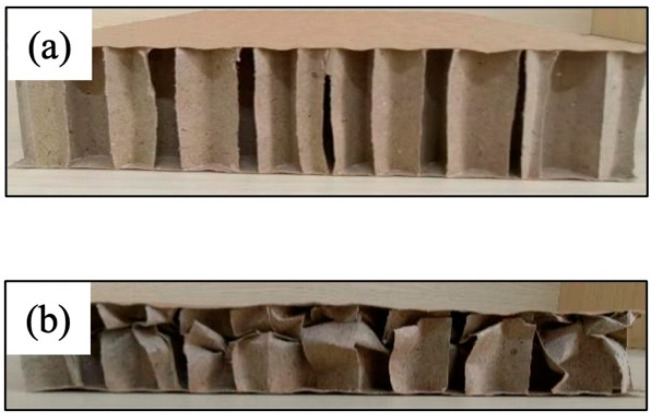
Comparison diagram of the honeycomb sandwich panel (**a**) before and (**b**) after impact.

The illustration in Figure 5 displays the comparison of the deformation of the honeycomb sandwich panel before and after the impact of the hammer. It is evident that under dynamic impact conditions, the face paper of the honeycomb sandwich panel remained undistorted, while the middle core paper layer underwent bending deformation to absorb energy. Each set of experiments was replicated five times under identical conditions. However, since the honeycomb sandwich panel was crushed after experiencing a single impact and lost its original cushioning performance, each honeycomb sandwich panel was utilized for only one test. After the dynamic compression experiment, under compression, the thickness of the honeycomb sandwich panel was gauged by using a Vernier caliper. This measurement was then compared with the thickness of the original sample and recorded as the depth of compression, signifying the compression displacement of the hammer.

**Figure 6:**
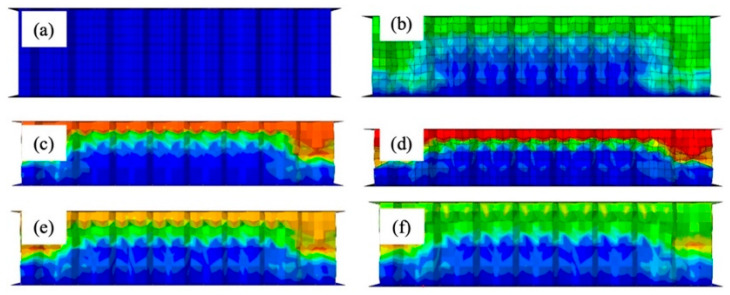
Impact deformation process: (**a**) 0 ms; (**b**) 3 ms; (**c**) 6 ms; (**d**) 10 ms; (**e**) 12 ms; (**f**) 15 ms.

Figure 6 shows the results of the dynamic impact finite element simulation on honeycomb sandwich panels, showing the deformation states at various time points. The figure’s color differences correspond to the different levels of stress, where blue indicates the lowest level of stress and red the highest level of stress.

The dynamic impact direction is aligned with the *Z*-axis, initiating elastic deformation in the specimen. Beyond the elastic limit, plastic deformation occurred. The core paper underwent gradual bending and wrinkling under the influence of velocity impact, forming a plastic hinge as the honeycomb cell wall folded layer by layer. With the progression of the impact, the wrinkles continued to fold downward, and the stress gradually increased. By the time t reached 10 ms, the stress reached its peak value, and the thickness of the honeycomb cardboard progressively decreased until the speed diminished to 0. At t = 12 ms, a rebound phenomenon was initiated, and the honeycomb sandwich panel experienced a brief period of small-scale rebound. Concurrently, the stress value gradually decreased. Figure 4 corresponds to the state at time t in Figure 5, which spans the interval between 0 ms and 10 ms.

### 3.2. Displacement–Time Curve

**Figure 7:**
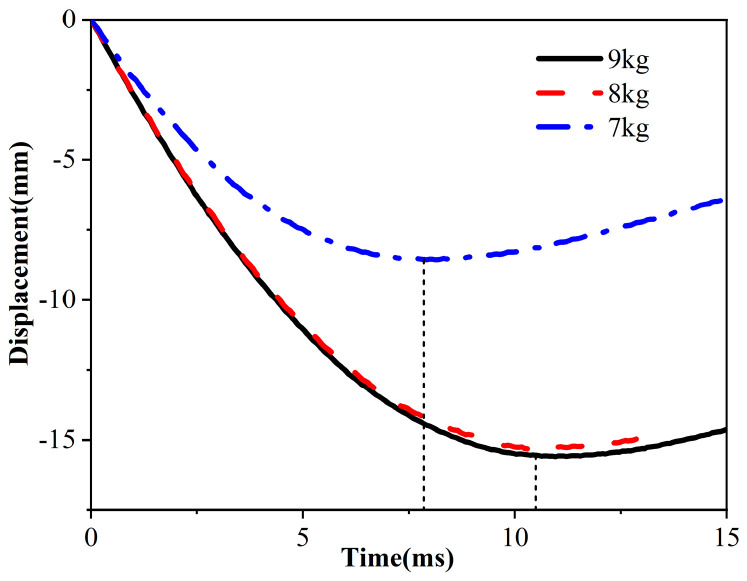
Displacement time curve.

The way in which sandwich panel structures deform and their ability to absorb energy are significantly impacted by the velocity and energy of the impacting force during a drop. In Figure 7, the curve depicts the displacement of the hammer over time, with the upward lifting direction designated as positive and the downward falling direction designated as unfavorable. The displacement curves of the three different masses of hammers exhibit a consistent trend: initial downward impact to maximum compression displacement followed by upward rebound. The experiment’s dynamic buffering machine, equipped with an inflation device to prevent the hammer dropping a second time on the material, resulted in a displacement curve that passed the peak moment, obscuring the precise observation of displacement value changes. The data analysis revealed that weights of 7 kg, 8 kg, and 9 kg impacting the honeycomb sandwich panel yielded displacement peaks of −8.52 mm, −15.2 mm, and −15.7 mm, respectively. The displacement value of 9 kg relative to 7 kg increased by 7.18 mm, while the displacement peak at 8 kg did not significantly differ from that at 9 kg. The error analysis suggested that the sample’s maximum impact load tolerance was between 7 kg and 8 kg. Beyond this threshold, the sample could not withstand excessive loads, leading to heightened damage.

### 3.3. Acceleration Compression Displacement Curve

**Figure 8:**
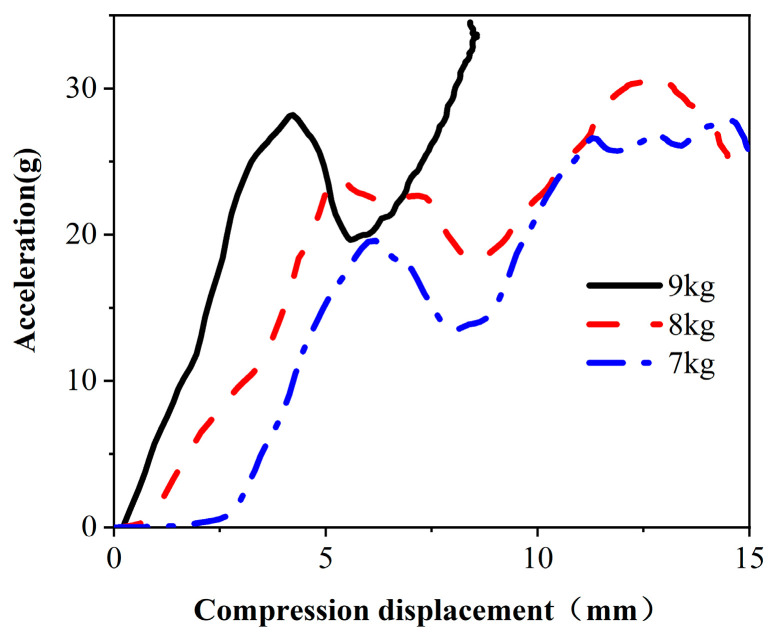
Acceleration compression displacement curve.

The dynamic cushioning performance of materials is usually characterized by peak acceleration. Figure 8 shows the acceleration compression displacement curve of a heavy hammer. Taking the 7 kg weight as an example, the maximum acceleration reached 19.5 g, the maximum acceleration corresponding to the 8 kg weight was 23.4 g, and the maximum acceleration corresponding to the 9 kg weight was 28.19 g. Compared to those of the 7 kg weight, the acceleration of the 8 kg and 9 kg weights increased by 20% and 44.5%, respectively. The slope of the acceleration curve of the 9 kg weight was also greater than that of the 7 kg and 8 kg weights, and the speed of change significantly increased. Under certain impact conditions, the panel received the transmitted impact energy, and the acceleration of the hammer gradually increased from zero. After reaching the peak, the core paper gradually absorbed the impact energy due to buckling, resulting in a gradual decrease in acceleration. Compared with those of traditional laminated panels, the energy absorption characteristics of honeycomb sandwich panels are significantly improved [28,29]. As the compression displacement increased, the honeycomb sandwich panel was gradually compacted, causing stress concentration and increasing the acceleration of the heavy hammer again. A time history analysis revealed that the 7 kg hammer reached its displacement peak at 7.8 ms, whereas the 9 kg hammer reached its peak at 10.85 ms. Smaller impact loads reach their peaks faster than larger loads, resulting in deeper compression of the cushioning material. An increase in the impact load prolongs the impact process, allowing the cushioning material to absorb the impact energy more fully. The data indicate that an increase in the impact load led to more significant compression displacement, increasing the deformation of the honeycomb panel. There was a maximum load-bearing range for the specimen, and surpassing this range rapidly increased damage. When designing and applying sandwich panel structures, it is imperative to consider and limit the level of impact load to ensure the required structural stability and performance in practical use.

### 3.4. Stress–Strain Curve

**Figure 9:**
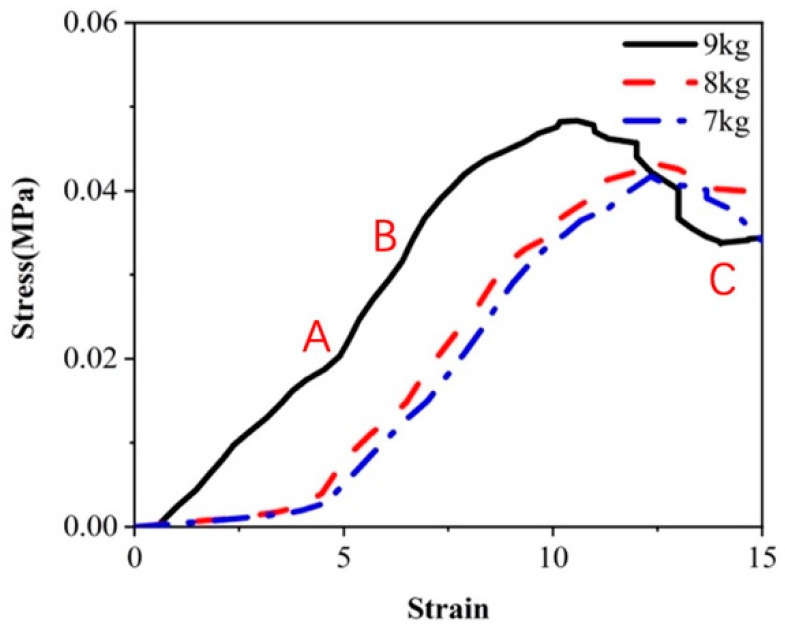
Stress–strain curve.

The stress–strain curve of honeycomb sandwich panels under dynamic impact is depicted in Figure 9. This is a commonly utilized method for characterizing material mechanical properties, enabling the observation of material behavior throughout the stress process. Elastic deformation occurred at the initial impact stage before point A, and the following formula is satisfied in this linear segment:

(10) σ=Eε

In the formula, *σ* (Pa) represents stress, *ε* represents strain, and *E* (Pa) represents the Young’s *E* modulus of the material, denoting the slope of the straight line, commonly known as Hooke’s law. Upon surpassing the elastic limit at point B, plastic deformation occurred in the honeycomb sandwich panel, and the core paper entered the yield stage before reaching the initial stress peak. The stress peaks corresponding to 9 kg, 8 kg, and 7 kg are 0.048 MPa, 0.043 MPa, and 0.04 MPa, respectively. The initial stress peak linearly rises with an increase in the hammer’s weight. Subsequently, due to the collapse and buckling of the internal structure of the core paper, the stress value initiates a decline. Upon reaching the yield limit at point C, slip lines in the core paper led to damage, resulting in a gradual decrease in cushioning performance. In the case of the 9 kg hammer, the initial stress peak was reached at a strain value of 10.8, while for 8 kg and 7 kg hammers, the initial stress peak was reached at strain values of 12.3 and 12.5, respectively. It is evident that the curve slope of the 9 kg hammer in the elastic stage is significantly larger than that of the 7 kg and 8 kg hammers. The increase in impact energy with the heavier hammer mass led to a more rapid impact load application to the cushioning material.

### 3.5. Static Stress–Cushioning Coefficient Curve

In the context of honeycomb sandwich panels, neglecting energy dissipation during the hammer drop process implies mechanical conservation. This concept asserts that, when the deformation of the honeycomb sandwich panel reaches its maximum, denoted as *X_m_* [m (or the strain reaches *ε_m_*)], the panel will experience the generation of the maximum static stress *σ_m_* (Pa) and peak acceleration *G_m_* (g). Following the law of energy conservation, the entire hammer’s kinetic energy undergoes conversion into the deformation energy of the honeycomb sandwich panel. Generally, when *H* >> *X_m_*, the equation obtained is as follows:

(11) $$AT\int_{0}^{\varepsilon_{m}}\sigma d\varepsilon =mgH$$

where *A* (m^2^) is the area of the cushioning material, *T* (m) is the thickness of the cushioning material, *m* (kg) is the mass of the hammer, *H* (m) is the height of the hammer drop, and the stress of the cushioning material under the gravity of a hammer is called the static stress, which is determined by *σ_st_*:

(12) $$\sigma_{st}=\frac{mg}{A}$$

The relationship between the maximum stress and static stress of the cushioning material under hammer impact is:

(13) $$\sigma_{m}=G_{m}\sigma_{st}$$

In the formula, *G_m_* (g) is the maximum impact acceleration of the hammer. Substitute Equations (2) and (3) into Equation (1) to obtain the relationship between the honeycomb cardboard thickness, drop height, and peak acceleration:

(14) $$T=C\frac{H}{G_{m}}$$

where:

(15) $$C=\frac{\sigma_{m}}{\int_{0}^{\varepsilon_{m}}\sigma d\varepsilon }=\frac{\frac{W}{A}G_{m}}{\frac{WH}{AT}}=\frac{\;G_{m}T}{H}$$

In the formula, the weight of the hammer, denoted as *W* (kg), determines the dynamic cushioning coefficient *C* based on the peak acceleration *G_m_* and static stress *σ_st_*. The shape of the *G_m_-σ_st_* curve illustrates the energy absorption efficiency of packaging cushioning materials against individual impacts, with a more minor curve change indicative of superior material protection for the product. The dynamic cushioning curve, derived from dynamic compression experimental data, has been established as the most practical basis for describing the shock absorption characteristics of cushioning packaging materials.

**Figure 10:**
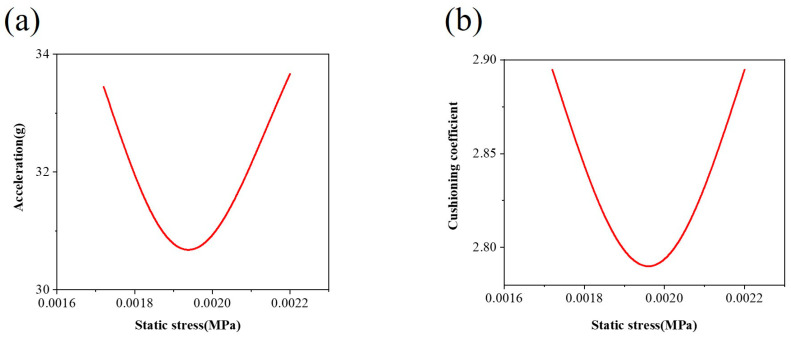
(**a**) Static stress acceleration curve and (**b**) static stress cushioning coefficient curve.

Figure 10 presents the peak acceleration static stress curve and dynamic cushioning curve, both characterized by a U-shaped configuration with an upward opening. As static stress increases, both the maximum acceleration and cushioning coefficient are observed to undergo a decrease followed by an initial increase. The concave point of the curve signifies the material’s maximum dynamic cushioning efficiency, aligning with observations in reference [14]. This point is where the actual application effect of the cushioning material is determined. Throughout this experiment, the contact area and drop height of the designated honeycomb sandwich panel remained constant. At the same time, three different weights were utilized to generate varying impact energies and static stresses.

For instance, when the weight was 8 kg, resulting in a static stress of 0.00196 MPa, the cushioning coefficient was observed to be the smallest at 2.79. A higher impact load per unit area corresponds to better cushioning performance, enabling the absorption of substantial impact energy during instantaneous impact. This performance was superior to that of the other two groups. The detailed values under different impact energies during the cushioning experiment are provided in Table 3.

## 4. Prediction of Cushioning Performance of Finite Element Models

### 4.1. Model Validation

**Figure 11:**
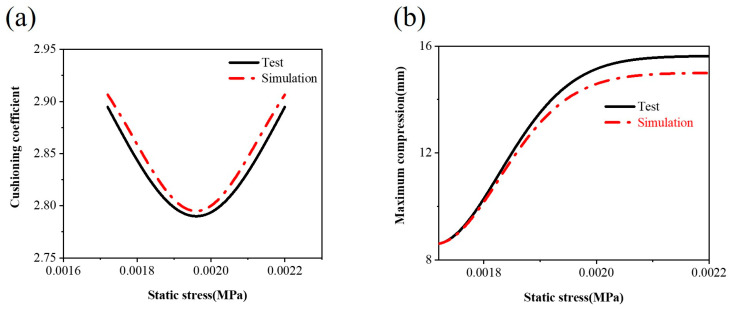
Comparison of the (**a**) static stress–cushioning coefficient and (**b**) static stress–maximum compression simulation experiments.

**Figure 12:**
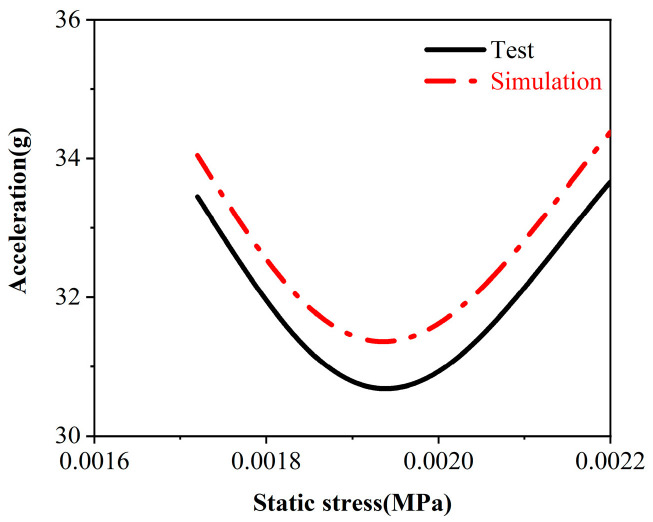
Comparison of the static stress acceleration simulation experiments.

A comparative analysis was conducted using experimental and simulation data to enhance the model’s credibility. Figure 11 and Figure 12 present the comparison charts for the cushioning coefficient, maximum compression, and acceleration derived from the experimental and simulation results. The overall trend is relatively consistent between the two datasets. The specific error analysis results for the experimental and simulation data are detailed in Table 4, where the error for the acceleration is 2.2%, the error for the maximum compression is 3.4%, and the error for the cushioning coefficient is 3.7%. All the error values fall within the 5% range, indicating that the simulation model accurately simulated the experimental conditions with high precision and reliability. This provides a dependable reference basis for further predictions and optimizations of structural models.

### 4.2. Prediction of the Cushioning Performance of Cell Edge Lengths

**Figure 13:**
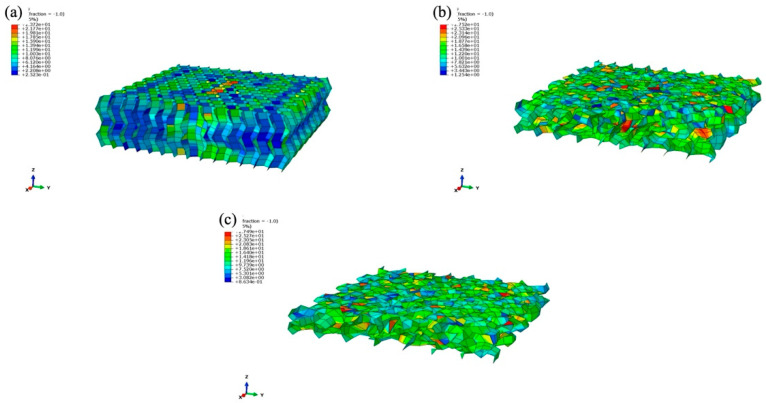
Stress nephograms of honeycomb sandwich panels with different cell edge lengths: (**a**) 4 mm; (**b**) 6 mm; (**c**) 8 mm.

In logistics processes, the honeycomb sandwich material serves as a representative cushioning and energy-absorbing protective material [30]. Building upon existing research, this study forecasts the dynamic cushioning performance of three distinct honeycomb cell lengths under various conditions and subsequently compares and analyzes the experimental outcomes. A stress nephogram depicting three types of honeycomb sandwich panel core paper with cell edge lengths of 4 mm, 6 mm, and 8 mm is illustrated in Figure 13. To better observe the force of the honeycomb core sheet, the upper and lower layers of the face sheet are concealed. The initial thickness of the sample was 30 mm, and it was compressed to final thicknesses of 26.82 mm, 15.39 mm, and 12.35 mm.

With an increase in cell length, the distance over which the honeycomb sandwich panel is compressed gradually expands. The nominal strains for honeycomb sandwich panels with cell edge lengths, arranged from smallest to largest, are 0.106, 0.487, and 0.583, respectively. The relative deformation between the top and bottom edges of a sample, divided by the sample’s initial height, is referred to as the nominal strain [31]. The sandwich panel featuring a cell length of 4 mm exhibited relatively little deformation energy, with the outer contour experiencing slight buckling. Conversely, panels with 6 mm and 8 mm cell side lengths displayed greater deformation energy, indicating that the core layer effectively absorbed impact energy, thereby reflecting superior cushioning performance. Notably, the middle layer of the core paper consistently exhibited higher stress concentrations.

**Figure 14:**
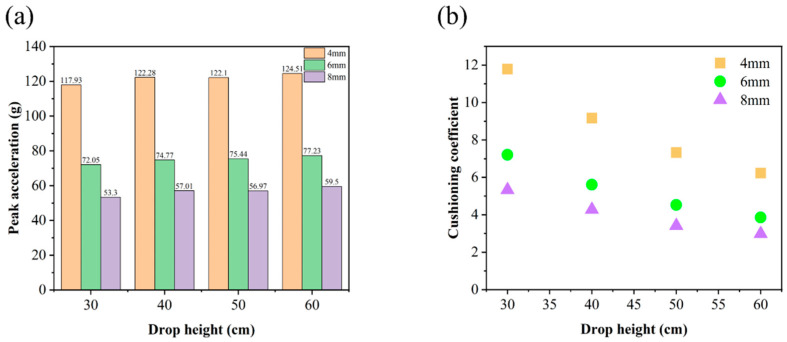
(**a**) Peak acceleration–drop height and (**b**) cushioning coefficient–drop height curves of honeycomb sandwich panels with different cell lengths.

As depicted in Figure 14, it is evident that the honeycomb sandwich panel with a cell edge length of 8 mm exhibited the smallest cushioning coefficient and peak acceleration among the three at varying heights. The peak acceleration was 53.4% lower than that of the honeycomb sandwich panel with a cell edge length of 4 mm and 24.3% lower than that of the honeycomb sandwich panel with a cell edge length of 6 mm, indicating a significant improvement in cushioning performance. Upon integrating the stress cloud map and the dynamic cushioning coefficient map for peak acceleration, the maximum stress for the 8 mm cell length was 0.0027 MPa, and the minimum cushioning coefficient was 2.98, which represents the reciprocal of the cushioning efficiency. Therefore, in selecting honeycomb sandwich panels for use as cushioning material, a panel with a smaller cushioning coefficient wherever possible should be selected to achieve material savings and the highest level of cushioning efficiency. These findings comprehensively demonstrate that the 8 mm honeycomb sandwich panel exhibits the most effective cushioning performance. In practice, a large amount of 8mm long honeycomb cardboard can be used, which provides a theoretical basis for reality. This kind of honeycomb cardboard can be used in logistics, aviation, and other fields.

### 4.3. Prediction of the Cushioning Performance of Face Sheet Thickness and Core Sheet Thickness

**Figure 15:**
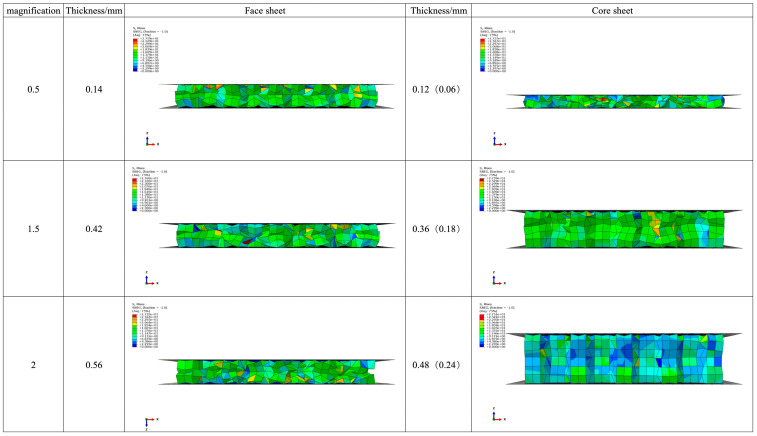
Stress nephograms of honeycomb sandwich panels with different thicknesses of face paper and core paper.

This chapter describes the cushioning properties of honeycomb sandwich panels with three distinct face paper thicknesses: 0.14 mm, 0.42 mm, and 0.56 mm, and honeycomb sandwich panels with three thicknesses of core paper: 0.12 mm, 0.36 mm, and 0.48 mm. The specific values are shown in Table 5. The original model data are presented in the second column. As the face paper thickness increased by a certain multiple, a linear decreasing trend in the cushioning coefficient was observed, ranging from 5.34 to 5.14. It can be clearly observed from the nephograms in Figure 15 that the thickness of the sandwich panel after testing was almost the same in all three cases, and the degree of buckling and failure of the core layer was also roughly the same, with the maximum stress values all being around 27.5 Pa. This difference is insignificant, indicating that changes in the face paper thickness of honeycomb sandwich panels have little effect on their overall cushioning effectiveness.

Similarly, the thickness of the core paper varied by a certain multiple. The cushioning coefficient increased from small to large with changes in the core paper thickness, i.e., 2.63, 5.32, 8.06, and 10.82. The values in parentheses represent the thickness of the single-layer honeycomb core paper, and the increment is notably larger than the changes in face paper thickness. When considering the stress nephogram in Figure 15, it becomes apparent that the compressed thickness of the honeycomb sandwich board decreased linearly, the degree of buckling of the core layer gradually decreased, and most of the sandwich panels with a thickness of 0.48 mm were still in the elastic stage, with only a few entering the yield stage, resulting in relatively poor cushioning performance, accompanied by a proportional decrease in the absorbed impact energy.

In summary, the thickness of the core paper is the primary factor influencing the cushioning performance of honeycomb sandwich panels, with a less significant correlation to the thickness of the face paper. When designing the honeycomb sandwich layer, it is advised to concentrate on the core layer and keep the thickness of the core paper within a narrow range in order to achieve the material’s best cushioning performance and avoid the honeycomb sandwich panel becoming overly hard, which could impair the panel’s ability to absorb impact energy and possibly damage the product.

## 5. Conclusions

Through simulation and experimentation, the reaction and mechanical characteristics of honeycomb sandwich panels under dynamic compression were investigated. Under various impact energies and cell lengths, the displacement, acceleration, stress–strain, and cushioning coefficient were examined. Dynamic compression experiments were recreated using modeling techniques to confirm the model’s accuracy, and the findings obtained agreed with the actual data. Using a simulation to estimate the cushioning performance of different structures, the following key findings were drawn:

(1) The experimental findings indicate that a reduction in the hammer weight will cause the honeycomb cardboard to sustain less damage, distortion, and collapse depth. Concurrently, the peak acceleration decreases as the hammer’s weight decreases. This is because less impact energy is produced by lighter hammers, which results in slower expansion rates, steeper acceleration curves, and more stiff collision processes. There is a maximum load-bearing range for the specimen; exceeding this limit will soon result in further damage. To guarantee the structural stability and performance needed for practical usage, the impact load level must be considered and limited when designing and implementing sandwich panel structures;
(2) According to the anticipated simulation results, cushioning effectiveness is improved as the cushioning coefficient progressively decreases with a relative increase in cell length. This suggests that shock energy absorption has improved, transforming this into deformation energy to minimize material consumption and increase cushioning effectiveness. Furthermore, it was discovered that altering the thickness of the surface paper had no effect on the deformation of the honeycomb sandwich panel, while altering the thickness of the core paper caused the deformation to vary significantly. It is evident that enhancing the ability of honeycomb sandwich panels to provide cushioning is significantly influenced by the thickness of the core paper;
(3) The experiment established a dynamic compression finite element model using the ABAQUS finite element numerical simulation approach. By simulating how a honeycomb paperboard deforms when subjected to dynamic loads at different speeds, the results were compared with experimental observations. The correctness and dependability of the established dynamic compression simulation model for honeycomb paperboard were confirmed by a deviation analysis, which showed that the difference between the simulation values and experimental findings was less than 5%. Additionally, a numerical simulation was used to assess the cushioning capacity of honeycomb sandwich panels with various cell lengths. The data indicated that the cushioning performance of honeycomb sandwich panels is closely related to that of the core paper, with the surface paper having no significant effect. Increasing the cell length appropriately within a certain range can also improve the cushioning performance.

## Figures and Tables

**Table 1:** Material properties of core sheet and face sheet.

Core Sheet Mass W/g/m^2^	Core Sheet Thickness T/mm	Yield Stress σ_γ_/MPa	Face Sheet Mass W/g/m^2^	Face Sheet Thickness T/mm	Yield Stress σ_γ_/MPa
80.00	0.12	8.70	200.00	0.28	7.80

**Table 2:** Material elastic parameters.

Elasticity Parameter	Face Sheet	Core Sheet
*E*_1_/MPa	3788.54	3516.72
*E*_2_/MPa	689.53	589.90
*E*_3_/MPa	18.90	17.58
*G*_12_/MPa	625.50	557.40
*G*_13_/MPa	68.90	63.92
*G*_23_/MPa	19.70	16.90
*ν*_12_	0.13	0.12
*ν*_13_	0.01	0.01
*ν*_23_	0.01	0.01

**Table 3:** Dynamic cushioning test data.

Test Parameters	9 kg	8 kg	7 kg
Size/mm	200 × 200 × 40	200 × 200 × 40	200 × 200 × 40
Drop height/cm	50	50	50
Static stress/MPa	0.0022	0.00196	0.00172
Peak acceleration/g	36.42	30.68	38.72
Maximum stress/KPa	48.23	43.25	42.22
Cushioning coefficient	2.91	2.79	3.10
Maximum compression/mm	15.63	15.44	8.59

**Table 4:** Comparison of the experimental and simulation errors.

Test Parameter	Test	Simulation	Deviation/%
Acceleration/g	30.68	31.36	2.2
Maximum Compression/mm	15.44	14.91	3.4
Cushioning coefficient	2.65	2.79	3.7

**Table 5:** Cushioning coefficient of face sheet and core sheet with different thicknesses.

Parameter	Thickness/mm/Cushioning Coefficient			
Face sheet	0.14/5.34	0.28/5.32	0.42/5.22	0.56/5.14
Core sheet	0.12 (0.06)/2.63	0.24 (0.12)/5.32	0.36 (0.18)/8.06	0.48 (0.24)/10.82

## Data Availability

The raw/processed data required to reproduce these findings cannot be shared at this time due to technical or time limitations.

## References

[1] Qi C., Jiang F., Yang S. (2021). Advanced honeycomb designs for improving mechanical properties: A review. Compos. Part B Eng..

[2] Zhang Y., Wang J., Wang C., Zeng Y., Chen T. (2018). Crashworthiness of bionic fractal hierarchical structures. Mater. Des..

[3] Karakoc A., Taciroglu E. (2016). Effects of morphology and topology on the effective stiffness of chiral cellular materials in the transverse plane. Adv. Mater. Sci. Eng..

[4] Yang X., Xi X., Pan Q., Liu H. (2019). In-plane dynamic crushing of a novel circular-celled honeycomb nested with petal-shaped mesostructure. Compos. Struct..

[5] Zhang X., An C., Shen Z., Wu H.-X., Yang W.-G., Bai J.-P. (2020). Dynamic crushing responses of bio-inspired re-entrant auxetic honeycombs under in-plane impact loading. Mater. Today Commun..

[6] Xue X., Zhang C., Chen W., Wu M., Zhao J. (2019). Study on the impact resistance of honeycomb sandwich structures under low-velocity/heavy mass. Compos. Struct..

[7] Han Q., Qin H., Han Z., Zhang J., Niu S., Sun Y., Shi S. (2020). Study on mechanical properties of multi-structure dactyl-inspired sandwich honeycomb with basalt fiber. Compos. Struct..

[8] Li Y., Wang F., Jia S., Ma X., Zhang Y. (2021). Numerical and experimental investigation of static four-point bending response of honeycomb sandwich structure: Failure modes and the effect of structural parameters. Fibers Polym..

[9] Fu Y., Sadeghian P. (2020). Flexural and shear characteristics of bio-based sandwich beams made of hollow and foam-filled paper honeycomb cores and flax fiber composite skins. Thin-Walled Struct..

[10] Chen Z., Yan N. (2012). Investigation of elastic moduli of Kraft Paper honeycomb core sandwich panels. Compos. Part B Eng..

[11] Wang D., Wang Z. (2008). Experimental investigation into the cushioning properties of honeycomb paperboard. Packag. Technol. Sci..

[12] Kmita-Fudalej G., Szewczyk W., Kotakowski Z. (2023). Bending Stiffness of Honeycomb Paperboard. Materials.

[13] Wang D., Bai Z. (2015). Mechanical property of paper honeycomb structure under dynamic compression. Mater. Des..

[14] Wang D. (2009). Impact behavior and energy absorption of paper honeycomb sandwich panels. Int. J. Impact Eng..

[15] Gu X., Wang J., Lu G., Pan L., Lu L. (2020). Modelling for the in-plane plateau stress of honeycomb paperboard based on the induce effect of face paper with honeycomb core. Int. J. Mech. Sci..

[16] Wang Z.-W., Yu P.E. (2010). Mathematical modelling of energy absorption property for paper honeycomb in various ambient humidities. Mater. Des..

[17] Zhao Y., Wang Y., Hao J. (2023). Study on mechanical properties of cellular structures with negative Poisson’s ratio based on the development of Abaqus plug-in tool. Compos. Struct..

[18] Xie S., Jing K. (2020). Mechanical properties of Nomex honeycomb sandwich panels under dynamic impact. Compos. Struct..

[19] Ma R., Li M. (2022). Geometry design and in-plane compression performance of novel origami honeycomb material. Thin-Walled Struct..

[20] Sun M., Diane W. (2022). Surface and honeycomb core damage in adhesively bonded aluminum sandwich panels subjected to low-velocity impact. Compos. Part B.

[21] Eipakchi H., Mahboubi N.F. (2022). Linear and nonlinear free vibration analysis of super light composite beams with honeycomb core layer and adjustable Poisson’s ratio using multiple-scale method. Acta Mech..

[22] Bohara R.P., Linforth S., Ghazlan A., Nguyen T., Remennikov A., Ngo T. (2021). Performance of an auxetic honeycomb core sandwich panelunder close-in and far-field detonations of high explosive. Compos Struct..

[23] (2016). Standard Practice for Damage Resistance Testing of Sandwich Constructions.

[24] (2000). Paper, Board and Pulps—Standard Atmosphere for Conditioning and Testing and Procedure for Monitoring the Atmosphere and Conditioning of Samples.

[25] Huang H., Nygards M. (2010). A simplified material model for finite element analysis of paperboard creasing. Nord. Pulp Pap. Res. J..

[26] Aktay L., Johnson A.F., Bernd H. (2008). Numerical modeling of honeycomb core crush behavior. Eng. Fract. Mech..

[27] Giglio M., Manes A., Gilioli A. (2012). Investigations on sandwich core properties through an experimental–numerical approach. Compos. Part B Eng..

[28] Yin S., Chen H., Wu Y., Li Y., Xu J. (2018). Introducing composite lattice core sandwich structure as an alternative proposal for engine hood. Compos. Struct..

[29] Wu X., Li Y., Cai W., Guo K., Zhu L. (2022). Dynamic responses and energy absorption of sandwich panel with aluminum honeycomb core under ice wedge impact. Int. J. Impact. Eng..

[30] Hong H., Hu M. (2021). Dynamic Mechanical Behavior of Hierarchical Resin Honeycomb by 3D Printing. Polymers.

[31] Zhou Y., Li Y. (2022). In-plane impact behavior of 3D-printed auxetic stainless honeycombs. Eng. Struct..

